# The Impact of Field Size on Positional and Possession Games: Variations in Physical Demands across Player Positions in Soccer Training

**DOI:** 10.5114/jhk/222302

**Published:** 2026-07-19

**Authors:** Jose Antonio Asian-Clemente, Bernardo Requena, Jose Vicente Beltran-Garrido, Piotr Żmijewski

**Affiliations:** 1 FSI Lab, Football Science Institute, Granada, Spain.; 2 Department of Sport Sciences, Universidad Pablo de Olavide, Seville, Spain.; 3 Physical Exercise and Performance Research Group, Department of Education Sciences, School of Humanities and Communication Sciences-Castellón, Universidad Cardenal Herrera-CEU, CEU Universities, Castellón de la Plana, Spain.; 4 Department of Biomedical Sciences, Faculty of Physical Education, Jozef Pilsudski University of Physical Education in Warsaw, Warsaw, Poland.

**Keywords:** football, training, male, games, running performance, small-sided games

## Abstract

This study investigated how pitch size and a task type (position vs. possession games) influence running demands and external load across player positions in soccer training. Twenty-five young professional soccer players (21.9 ± 1.9 years) participated in 36 training sessions during the 2021–2022 season. Position and possession games were conducted on three pitch sizes (small, medium, large). Players wore GPS devices to measure total distance covered (DC), peak speed, and acceleration/deceleration metrics (low-intensity < ±3 m•s⁻^2^ and high-intensity > ±3 m•s⁻^2^). Mixed models assessed the effects of pitch size and a task type on these metrics and positional differences. Possession games elicited higher total DC than position games across all sizes studied (p < 0.005). In the large format, the peak speed was higher in possession than in position games (p < 0.001). On small pitches, possession games resulted in greater low-intensity accelerations/decelerations (ACC_<3_/DEC_<3_), but fewer high-intensity accelerations/decelerations (ACC_>3_/DEC_>3_) and maximal deceleration compared to position games (p < 0.05). Positional analysis revealed that central defenders and fullbacks reached greater total DC across all three formats of possession games (p < 0.05). Central-midfielders and forwards recorded higher peak speeds in the large possession game (p ≤ 0.01). Wide-midfielders achieved fewer ACC_<3_ and DEC_<3_ events in position games during the small format, while fullbacks performed fewer ACC_<3_ but more ACC_>3_ in the same format during position games (p ≤ 0.05). Field size and the game format significantly affect external load in soccer. Possession games require greater total DC regardless of size and higher peak speeds on larger fields, while position games involve more high-intensity actions, reflecting players’ tactical roles.

## Introduction

Load monitoring is fundamental to reducing the risk of injury, optimizing performance, and preventing non-functional overtraining in sports (Clemente et al., 2019; Halson, 2014). In soccer, this monitoring process has gained significant relevance as it allows for the adjustment and prescription of load volume and intensity for players (Sedeaud et al., 2020), achieving suitable physical stimuli during training sessions (Clemente et al., 2020). Similarly, monitoring the load can help to gain an overall understanding of how the training stimuli differ from match demands (Stevens et al., 2017). This comparison between training and competitive play is very important because it allows for determining whether the selected training sessions elicit similar, greater or lower loads compared to match-play (Asian-Clemente et al., 2022a).

In addition to controlling the stimulus magnitude, an aspect currently considered essential is translating match demands into positional drills (Ade et al., 2021) to enhance players’ performance. Small-sided games are widely used in football training as an integrated approach to developing technical, tactical, and physical capacities. Their effectiveness is achieved through the systematic manipulation of task constraints, such as the number of players, pitch dimensions, and game rules, among other variables (Beato et al., 2026; de Dios-Álvarez et al., 2025; Gonzalez-Rodenas et al., 2025). With this approach, a type of the task known as 'position games' has been developed in soccer. Position games are small-sided games where players perform according to specific positional roles, aiming to replicate scenarios similar to those encountered in competitive play (Asian-Clemente et al., 2024a, 2025). Although position games are widely utilised in soccer training at all levels, they remain insufficiently explored in the literature. A recent study comparing the running demands of position games of various sizes to official matches found that soccer players covered greater distances at speeds >21 km•h^-1^, reached higher peak speeds, and experienced more low-intensity accelerations and decelerations, as well as peak acceleration and deceleration during competition. However, training drills elicit a higher frequency of high-intensity accelerations and decelerations (Asian-Clemente et al., 2025). Similarly, another recent study analysing the running demands of position games and possession games found that, in the latter, players reached greater total distance covered (DC), peak speeds, and experienced a greater player load, but performed fewer high-intensity accelerations and decelerations than in position games (Asian-Clemente et al., 2024a). That study also compared the same position game played in different game sizes, finding that when the relative area per player increased, players covered greater distances at speeds >21 km•h^-1^, experienced higher player loads, and performed more maximal accelerations and decelerations; however, this also resulted in reduced demands for lower-intensity accelerations and decelerations (Asian-Clemente et al., 2024a).

Considering that small-sided games, due to their similarity to competition and their efficiency, are currently regarded in the literature as training tasks that best simulate competitive demands for training soccer players (Asian-Clemente et al., 2022b; Olthof et al., 2018), continuing to study the relationship between position games and possession sided games is necessary. Given that the playing area is reported as one of the main variables influencing running demands during small-sided games (Bujalance-Moreno et al., 2019; Sarmento et al., 2018), comparing positional games and possession-based games of varying sizes is crucial for gaining a deeper understanding of these novel training tasks. Similarly, since both the position occupied and tactical behaviour are key factors significantly affecting the running demands of soccer players (Asian-Clemente et al., 2022b; Lorenzo-Martinez et al., 2021; Paul et al., 2015; Thoseby et al., 2023), it is essential to study the differences in individual requirements by position between positional games and possession-based games of different sizes. Accordingly, the objectives of this study were: (1) to analyse the running demands of positional games and possession-based games played on various pitch sizes, and (2) to evaluate how individual external load requirements would differ across player positions in these scenarios.

## Methods

### Participants

Twenty-five young soccer players of the second team of a professional Spanish first division team participated in this study (age: 21.9 ± 1.9 years; body height: 177.9 ± 5.2 cm; body mass: 75.5 ± 4.8 kg; % body fat (Faulkner): 11.1 ± 1.4%). A total of 656 individual data points of outfield players (goalkeepers excluded) were included in the analysis. This study involved retrospective analysis of data collected as part of the club's routine training load monitoring program, which all players underwent as a condition of employment. According to institutional guidelines and consistent with the framework outlined by Winter and Maughan (2009), formal ethics committee approval was not required for such observational analyses of existing training data. However, the study was conducted in full accordance with the Declaration of Helsinki. All players were informed about the data collection procedures and provided written consent for the potential use of anonymised data for research purposes. The institutional data protection officer approved the use of these data for research.

### Measures

To assess the external load on soccer players during matches and positional games, all players were equipped with GPS devices (WIMU Pro, RealTrack Systems, Almería, Spain) featuring a sampling rate of 10 Hz. This device's validity and reliability in collecting time-motion variables have been thoroughly analysed and established as suitable instruments for soccer purposes (Bastida Castillo et al., 2017, 2018). Various metrics were recorded, including total DC, peak speed, maximal accelerations and decelerations, as well as accelerations and decelerations below ±3 m•s^−2^ (Acc_<3_; Dec_<3_, respectively) and those above ±3 m•s^−2^ (Acc_>3_; Dec_>3_, respectively). These specific variables had been widely utilised in prior literature (Asian-Clemente et al., 2021, 2024a, 2025).

### Design and Procedures

Throughout the 2021–2022 season, a descriptive design of 36 training sessions to compare the external load of two types of small-sided games (position and possession games) were analysed. In a format of 9 vs. 9 + 2 floaters, players participated in three position and possession games categorised depending on their pitch dimension as small (position games = 33.8 ± 2.9 m x 31.0 ± 3.7 m, relative area per player = 50.8 ± 6.6 m^2^ and possession games = 33.1 ± 2.6 m x 31.3 ± 2.7 m, relative area per player = 46.1 ± 21.0 m^2^ and), medium (position games = 42.0 ± 1.9 m x 38.7 ± 2.1 m, relative area per player = 80.5 ± 4.6 m^2^ and possession games = 40.7 ± 1.0 m x 40.0 ± 3.6 m, relative area per player = 80.5 ± 4.6 m^2^) and large (position games = 48.8 ± 5.2 m x 46.5 ± 4.8 m, relative area per player = 115.9 ± 25.2 m^2^ and possession games = 48.5 ± 5.7 m x 46.8 ± 4.5 m, relative area per player = 115.0 ± 25.2 m^2^). These games were played in a randomised sequence on different days throughout the 36-week regular season, aligning with the tactical and physical demands of the micro-cycle. Each game was analysed six times during this period.

The key distinction between the formats was that in possession games, players moved freely without a fixed positional role, while in position games, they had to adhere to specific roles assigned within a 4-2-3 formation (a variation of the 4-3-3 setup with 9 players per team), based on their predefined positions. This system of play was the most frequently used by the team during the competition. Similarly, the floaters’ behavior differed between drills; in possession games, they could move freely across the field, whereas in position games, they were positioned on the sidelines, areas typically occupied by either goalkeepers or strikers, depending on which team had possession. In all formats, the tasks were structured to maintain ball possession for as long as possible.

Each session began with a uniform 20-min warm-up that included running, ball possession activities, and dynamic stretching exercises before moving on to the specific drills. Tasks were conducted in a continuous format for 8 min, permitting a maximum of two touches per player. Coaches participated actively by providing verbal support, constructive feedback, and ensuring the quick reintroduction of the ball whenever it left the playing area.

The first objective of the study involved analyzing the external load of players to compare the demands of both tasks within the same playing area (small position games vs. small possession games; medium position games vs. medium possession games; large position games vs. large possession games). For the second objective, players were categorized into six distinct positions: central-defenders (CDs), fullbacks (FBs), defensive-midfielders (DMs), offensive-midfielders (OMs), wide-midfielders (WMs) and forwards (Fs), comparing the same position in both designed tasks and three field sizes.

### Statistical Analysis

To confirm the data normality of each dataset, the Kolmogorov-Smirnov test, the Q-Q plot of residuals and the random coefficients histogram were used. Data not following a normal distribution were transformed before further analysis (Field, 2017). Mixed model analyses were used to compare the effects of space’s dimensions (i.e., small, medium, large) of the position and possession tasks on the dependent variables. The model used for each dependent variable was with size and the type of the task as independent fixed factors and random intercepts on the individual player. A log-likelihood ratio test was used to assess the goodness of fit of the models. To assess between-tasks differences at each space’s dimension, simple effects were calculated with the type of the task as the simple effects variable and space’s dimension as the moderator variable. Mixed model analyses were used to compare the effects of the players’ position (CD, FB, CM, WM, OM and F) and space’s dimensions (small, medium and large) of the position and possession games on the dependent variables. The model used for each dependent variable was with the player’s position, size and the type of the task as independent fixed factors and random intercepts on the individual player. A log-likelihood ratio test was used to assess the goodness of fit of the models. To assess between-tasks differences at each space’s dimension, simple effects were calculated with the type of the task as a simple effects variable, space’s dimension as a moderator variable and the player’s position as a breaking variable. Standardized mean difference Cohen’s *d* effect sizes were obtained and were interpreted as: <0.2 = trivial; 0.2–0.59 = small; 0.6–1.19 = moderate; 1.2–1.99 = large; ≥2.0 = very large (Hopkins et al., 2009). Statistical significance was set at α < 0.05. Unless otherwise stated, all values are presented as estimated marginal mean (SE) or estimated marginal mean and the 95% CI. The data analysis was performed using JAMOVI for Mac (version 2.6.17; The jamovi Project, 2022) and the jamovi module GAMLj: General analyses for linear models (Gallucci, 2019).

## Results

**Figure 1:**
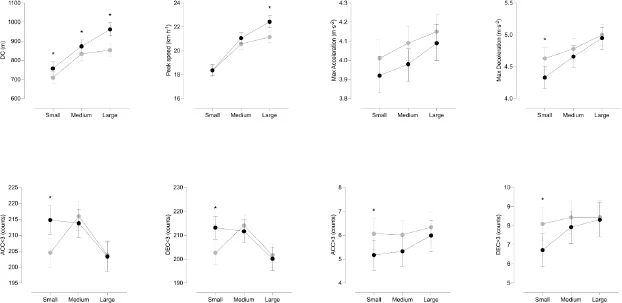
Comparison of the external load of position and possession games. Gray line: Position games; Black line: Possession games; DC = Distance covered; Max = Maximal; ACC_<3_ = Accelerations lower than 3 m•s^−2^; DEC_<3_ = decelerations lower than −3 m•s^−2^; ACC_>3_ = Accelerations higher than 3 m•s^−2^; DEC_>3_ = Decelerations higher than −3 m•s^−2^; Small: small size of the task’s space; Medium: medium size of the task’s space; Large: large size of the task’s space; * p ≤ 0.05 significantly different from the position task

**Figure 2:**
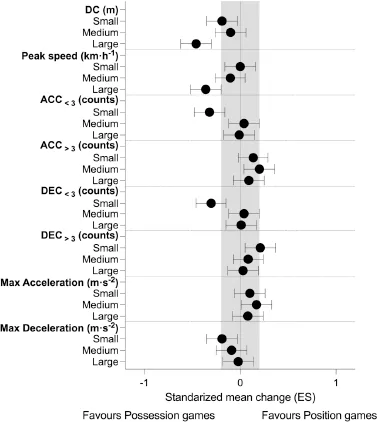
Effect sizes (ES) comparison of position and possession games. DC = Distance covered; Max = Maximal; ACC_<3_ = Accelerations lower than 3 m•s^−2^; DEC_<3_ = decelerations lower than −3 m•s^−2^; ACC_>3_ = Accelerations higher than 3 m•s^−2^; DEC_>3_ = Decelerations higher than −3 m•s^−2^; Small: small size of the task’s space; Medium: medium size of the task’s space; Large: large size of the task’s space

Descriptive data of the position and possession games are presented in Table 1 as well as in Figures 1 and 2. Small possession games showed significantly higher total DC (MD ± SE = 48.41 ± 14.38 m, *p* < 0.001, *d* = −0.19), ACC_<3_ (MD ± SE = 10.31 ± 2.47 counts, *p* < 0.001, *d* = −0.32) and DEC_<3_ (MD ± SE = 10.54 ± 2.85 counts, *p* < 0.001, *d* = −0.30), but significantly lower maximal deceleration (MD ± SE = 0.26 ± 0.11 m•s^−2^, *p* = 0.018, *d* = 0.19), less ACC_>3_ (MD ± SE = −0.89 ± 0.38 counts, *p* = 0.022, *d* = 0.14) and DEC_>3_ (MD ± SE = −1.37 ± 0.50 counts, *p* = 0.006, *d* = 0.21) than small position games. During medium possession games, players recorded higher total DC (MD ± SE = 39.69 ± 14.07 m, *p* = 0.005, *d* = −0.10) than in medium position games. For their part, large possession games resulted in significantly greater total DC (MD ± SE = 108.32 ± 14.62 m, *p* < 0.001, *d* = −0.46) and peak speed (MD ± SE = 1.27 ± 0.30 km•h^−1^, *p* < 0.001, *d* = −0.36) compared to large position games.

**Table 1:** External load of position and possession games based on their pitch size.

Variable		Task type	Small	Medium	Large
Total DC (m)		Position	709.47 ± 16.69	833.44 ± 16.43	853.58 ± 16.65
		Possession	757.88 ± 16.55*	873.13 ± 16.46*	961.90 ± 17.04*
Peak speed (km•h^−1^)		Position	18.35 ± 0.25	20.57 ± 0.24	21.16 ± 0.24
		Possession	18.37 ± 0.24	21.06 ± 0.24	22.43 ± 0.25*
Max Acceleration (m•s^−2^)		Position	4.01 ± 0.05	4.09 ± 0.05	4.15 ± 0.05
		Possession	3.92 ± 0.05	3.98 ± 0.05	4.09 ± 0.05
Max Deceleration (m•s^−2^)		Position	4.63 ± 0.09	4.78 ± 0.08	5.00 ± 0.09
		Possession	4.33 ± 0.09*	4.66 ± 0.08	4.95 ± 0.09
ACC_<3_ (counts)		Position	204.48 ± 2.31	215.95 ± 2.28	203.80 ± 2.30
		Possession	214.79 ± 2.26*	213.77 ± 2.25	203.31 ± 2.34
ACC_>3_ (counts)		Position	6.06 ± 0.33	6.02 ± 0.32	6.34 ± 0.33
		Possession	5.17 ± 0.32*	5.33 ± 0.32	5.99 ± 0.33
DEC_<3_ (counts)		Position	202.67 ± 2.49	214.10 ± 2.44	201.63 ± 2.47
		Possession	213.20 ± 2.43*	211.65 ± 2.42	200.17 ± 2.51
DEC_>3_ (counts)		Position	8.09 ± 0.44	8.44 ± 0.43	8.45 ± 0.44
		Possession	6.72 ± 0.43*	7.93 ± 0.43	8.31 ± 0.44

DC = distance covered; Max = Maximal; ACC_<3_ = Accelerations lower than 3 m•s^−2^; DEC_<3_ = Decelerations lower than −3 m•s^−2^; ACC_>3_ = Accelerations higher than 3 m•s^−2^; DEC_>3_ = Decelerations higher than −3 m•s^−2^; Small: small size of the task’s space; Medium: medium size of the task’s space; Large: large size of the task’s space. * p ≤ 0.05 significantly different from the position task

**Figure 3:**
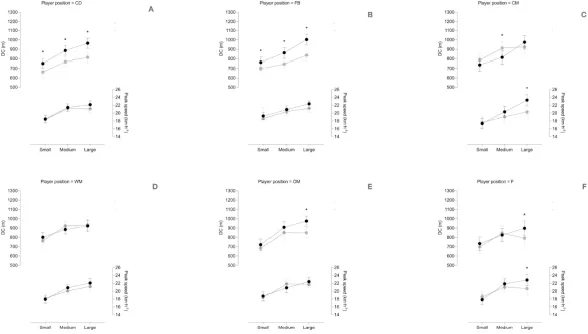
Distance covered and peak speed comparison for each position in position games and possession games. Gray line: Position games; Black line: Possession games; CD = Central-Defenders; FB = Fullbacks, DM = Defensive-Midfielders, OM = Offensive-Midfielders; WM = Wide-midfielders, F = Forwards; DC = distance covered; Small: small size of the task’s space; Medium: medium size of the task’s space; Large: large size of the task’s space. * p ≤ 0.05 significantly different from the position task

**Figure 4:**
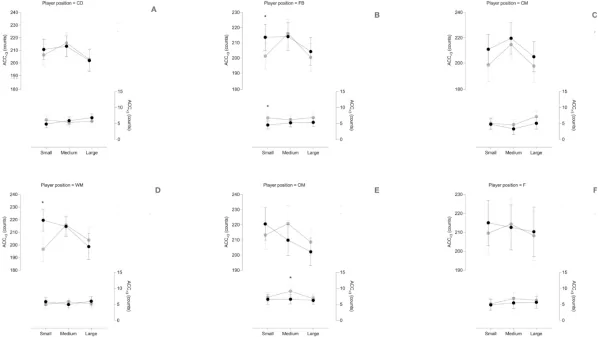
Maximal acceleration and deceleration comparison for each position in position and possession games. Gray line: Position games; Black line: Possession games; CD = Central defenders; FB = Fullbacks, DM = Defensive midfielders, OM = Offensive midfielders; WM = Wide midfielders, F = Forwards; Max = Maximal; Small: small size of the task’s space; Medium: medium size of the task’s space; Large: large size of the task’s space. * p ≤ 0.05 significantly different from the position task

**Figure 5:**
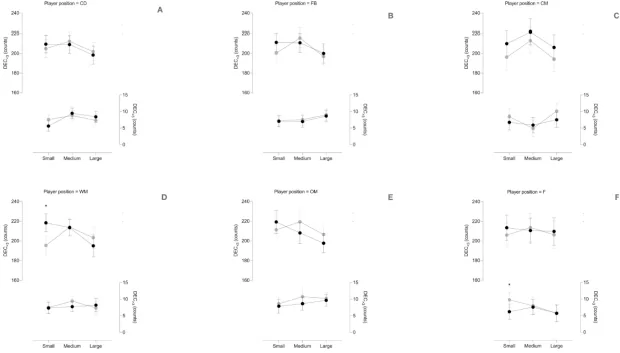
Accelerations comparison for each position in position and possession games. Gray line: Position games; Black line: Possession games; CD = Central defenders; FB = Fullbacks; DM = Defensive midfielders; OM = Offensive midfielders; WM = Wide midfielders; F = Forwards; ACC_<3_ = Accelerations lower than 3 m•s^−2^; ACC_>3_ = Accelerations higher than 3 m•s^−2^; Small: small size of the task’s space; Medium: medium size of the task’s space; Large: large size of the task’s space. * p ≤ 0.05 significantly different from the position task

The positional analysis comparing the position and possession games is shown in Table 2 and Figures 3–5 and 7.

**Table 2:** Comparison of each role position during the position and possession games.

		Position						Possession		
Variable	Position	Small	Medium	Large		Small			Medium	Large
Total DC (m)	CD	662.6 ± 26.0	769.4 ± 24.3	814.0 ± 26.3		753.1 ± 25.8*			886.5 ± 25.8*	963.7 ± 27.0*
	FB	697.5 ± 25.8	746.9 ± 26.9	836.6 ± 27.3		764.6 ± 26.5*			862.4 ± 27.4*	1003.0 ± 28.8*
	CM	785.6 ± 36.4	911.3 ± 34.8	923.7 ± 35.6		738.2 ± 34.5			813.9 ± 35.4*	974.0 ± 36.6
	WM	766.0 ± 29.0	922.1 ± 23.7	934.2 ± 29.7		801.4 ± 25.9			885.3 ± 24.1	923.5 ± 30.8
	OM	683.7 ± 28.4	851.5 ± 34.5	850.1 ± 26.0		721.7 ± 31.6			908.4 ± 30.1	975.2 ± 27.3*
	F	698.3 ± 34.4	845.7 ± 41.0	792.7 ± 40.1		733.8 ± 36.3			826.0 ± 36.2	898.1 ± 40.1*
Peak Speed (km•h^−1^)	CD	18.36 ± 0.49	21.18 ± 0.45	21.06 ± 0.49		18.50 ± 0.47			21.43 ± 0.47	22.14 ± 0.49
	FB	18.62 ± 0.48	20.42 ± 0.50	21.23 ± 0.51		19.26 ± 0.49			20.91 ± 0.52	22.36 ± 0.51
	CM	17.61 ± 0.72	19.08 ± 0.70	20.31 ± 0.71		17.36 ± 0.68			20.33 ± 0.71	23.30 ± 0.70*
	WM	18.20 ± 0.58	20.06 ± 0.44	21.22 ± 0.59		17.94 ± 0.50			20.89 ± 0.45	22.07 ± 0.59
	OM	18.41 ± 0.56	21.80 ± 0.70	21.79 ± 0.49		18.77 ± 0.63			20.86 ± 0.60	22.42 ± 0.53
	F	18.56 ± 0.64	21.07 ± 0.74	20.69 ± 0.72		17.85 ± 0.67			21.86 ± 0.67	22.83 ± 0.72*
Max Acceleration (m•s^−2^)	CD	4.14 ± 0.10	4.09 ± 0.09	3.97 ± 0.10		3.96 ± 0.09			4.13 ± 0.09	3.97 ± 0.10
	FB	3.97 ± 0.10	4.10 ± 0.10	4.19 ± 0.10		3.98 ± 0.10			3.91 ± 0.11	4.09 ± 0.10
	CM	4.00 ± 0.15	3.95 ± 0.15	4.39 ± 0.14		4.02 ± 0.14			3.74 ± 0.14	4.23 ± 0.14
	WM	3.89 ± 0.12	4.03 ± 0.09	3.90 ± 0.12		3.90 ± 0.10			3.89 ± 0.09	4.17 ± 0.12
	OM	4.05 ± 0.12	4.22 ± 0.15	4.27 ± 0.10		3.84 ± 0.12			4.14 ± 0.12	4.11 ± 0.11
	F	3.99 ± 0.13	4.25 ± 0.15	4.33 ± 0.14		3.84 ± 0.13			3.99 ± 0.13	4.10 ± 0.15
Max Deceleration (m•s^−2^)	CD	4.91 ± 0.18	5.36 ± 0.17	5.32 ± 0.19		4.56 ± 0.18			4.91 ± 0.18*	4.90 ± 0.18
	FB	4.62 ± 0.19	4.66 ± 0.19	4.76 ± 0.19		4.74 ± 0.19			4.62 ± 0.20	4.99 ± 0.19
	CM	4.48 ± 0.28	4.33 ± 0.27	4.92 ± 0.26		4.08 ± 0.26			4.39 ± 0.26	5.00 ± 0.26
	WM	4.57 ± 0.21	4.51 ± 0.17	4.84 ± 0.22		4.13 ± 0.19			4.60 ± 0.17	4.80 ± 0.23
	OM	4.61 ± 0.22	5.17 ± 0.27	5.29 ± 0.19		4.43 ± 0.23			4.73 ± 0.22	5.21 ± 0.20
	F	4.42 ± 0.25	4.77 ± 0.29	4.92 ± 0.27		4.46 ± 0.25			4.63 ± 0.26	5.02 ± 0.29
ACC_<3_ (counts)	CD	206.4 ± 4.3	215.8 ± 4.1	203.0 ± 4.4		210.8 ± 4.2			213.3 ± 4.2	202.0 ± 4.4
	FB	201.3 ± 4.4	216.2 ± 4.7	200.5 ± 4.7		213.7 ± 4.3*			214.2 ± 4.6	204.3 ± 4.6
	CM	198.5 ± 6.3	214.7 ± 6.0	197.6 ± 6.2		210.9 ± 6.0			219.6 ± 6.3	205.1 ± 6.1
	WM	196.7 ± 5.0	215.9 ± 4.0	204.0 ± 5.1		219.6 ± 4.4*			214.7 ± 4.0	198.7 ± 5.3
	OM	213.2 ± 4.9	220.8 ± 6.0	208.6 ± 4.4		220.5 ± 5.5			209.9 ± 5.2	202.2 ± 4.6
	F	209.6 ± 5.8	214.4 ± 6.8	208.2 ± 6.6		215.1 ± 6.1			212.6 ± 6.0	210.3 ± 6.6
ACC_>3_ (counts)	CD	6.15 ± 0.63	5.30 ± 0.57	5.71 ± 0.63		4.81 ± 0.60			5.87 ± 0.61	6.80 ± 0.63
	FB	6.74 ± 0.64	6.15 ± 0.64	6.87 ± 0.66		4.52 ± 0.64*			5.22 ± 0.66	5.35 ± 0.66
	CM	5.05 ± 0.92	4.56 ± 0.89	7.18 ± 0.90		4.75 ± 0.87			3.31 ± 0.90	5.06 ± 0.94
	WM	5.06 ± 0.73	5.83 ± 0.57	5.28 ± 0.74		5.85 ± 0.64			4.99 ± 0.58	6.01 ± 0.75
	OM	7.26 ± 0.71	9.14 ± 0.89	6.79 ± 0.67		6.59 ± 0.80			6.62 ± 0.76*	6.28 ± 0.67
	F	5.19 ± 0.85	6.83 ± 1.00	6.32 ± 0.94		4.86 ± 0.87			5.47 ± 0.86	5.66 ± 0.94
DEC_<3_ (counts)	CD	204.9 ± 4.7	212.3 ± 4.4	201.8 ± 4.7		209.4 ± 4.5			209.0 ± 4.5	198.4 ± 4.7
	FB	200.4 ± 4.7	215.6 ± 4.9	196.9 ± 4.9		211.2 ± 4.7			210.8 ± 4.9	200.0 ± 4.9
	CM	196.2 ± 6.7	213.0 ± 6.5	194.3 ± 6.6		209.9 ± 6.4			221.5 ± 6.6	206.0 ± 6.5
	WM	195.4 ± 5.3	214.1 ± 4.2	203.3 ± 5.5		218.4 ± 4.7*			213.4 ± 4.2	195.0 ± 5.7
	OM	211.2 ± 5.2	219.4 ± 6.5	206.4 ± 4.7		219.2 ± 5.9			208.1 ± 5.6	197.7 ± 5.0
	F	206.0 ± 6.2	213.7 ± 7.3	206.3 ± 7.1		213.5 ± 6.5			210.6 ± 6.5	209.7 ± 7.1
DEC_>3_ (counts)	CD	7.59 ± 0.84	8.79 ± 0.77	7.34 ± 0.84		5.61 ± 0.81			9.46 ± 0.82	8.40 ± 0.85
	FB	7.27 ± 0.85	7.55 ± 0.87	9.03 ± 0.88		7.05 ± 0.85			6.98 ± 0.89	8.65 ± 0.89
	CM	8.44 ± 1.22	4.91 ± 1.23	10.12 ± 1.20		6.72 ± 1.15			5.90 ± 1.19	7.50 ± 1.18
	WM	7.51 ± 0.97	9.43 ± 0.76	7.34 ± 0.99		7.34 ± 0.85			7.76 ± 0.76	8.20 ± 1.00
	OM	8.59 ± 0.94	10.74 ± 1.17	10.23 ± 0.87		7.88 ± 1.06			8.66 ± 1.01	9.66 ± 0.90
	F	9.75 ± 1.11	7.99 ± 1.31	5.73 ± 1.31		6.19 ± 1.17*			7.56 ± 1.17	5.71 ± 1.27

DC = distance covered; Max = Maximal; ACC_<3_ = Accelerations lower than 3 m•s^−2^; DEC_<3_ = Decelerations lower than −3 m•s^−2^; ACC_>3_ = Accelerations higher than 3 m•s^−2^; DEC_>3_ = Decelerations higher than −3 m•s^−2^; CD = Central defender; FB = Fullback; CM = Central midfielder; WM = Wide midfielder; OM = Offensive midfielder; F = Forward. Small: small size of the task’s space; Medium: medium size of the task’s space; Large: large size of the task’s space. * p ≤ 0.05 significantly different from the same size of position game

**Figure 6:**
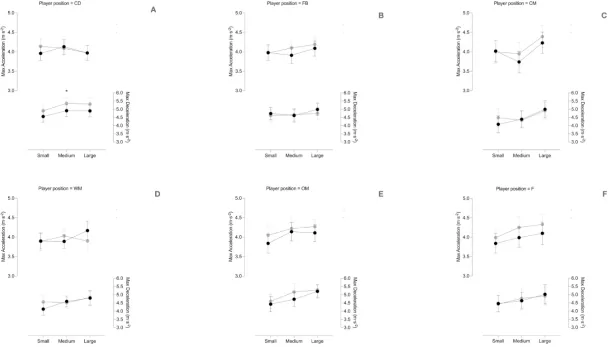
Decelerations comparison for each position in position and possession games. Gray line: Position games; Black line: Possession games; CD = Central defenders; FB = Fullbacks; DM = Defensive midfielders; OM = Offensive midfielders; WM = Wide midfielders; F = Forwards; DEC_<3_ = decelerations lower than −3 m•s^−2^; DEC_>3_ = Decelerations higher than −3 m•s^−2^; Small: small size of the task’s space; Medium: medium size of the task’s space; Large: large size of the task’s space. * p ≤ 0.05 statistically significantly different from the position task

**Figure 7:**
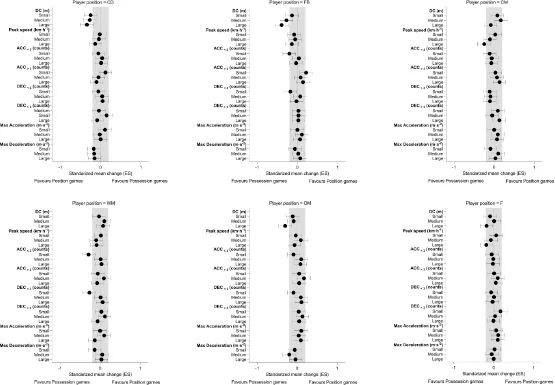
Effect sizes (ES) comparison of position and possession games for each playing position. CD = Central defenders; FB = Fullbacks; DM = Defensive midfielders; OM = Offensive midfielders; WM = Wide midfielders, F = Forwards; DC = Distance covered; Max = Maximal; ACC_<3_ = Accelerations lower than 3 m•s^−2^; DEC_<3_ = decelerations lower than −3 m•s^−2^; ACC_>3_ = Accelerations higher than 3 m•s^−2^; DEC_>3_ = Decelerations higher than −3 m•s^−2^; Small: small size of the task’s space; Medium: medium size of the task’s space; Large: large size of the task’s space

### Small Dimensions

CD players exhibited higher values of total DC (MD ± SE = 90.53 ± 29.19 m; *p* = 0.002, *d* = −0.25) during possession games compared to position games. FB players showed higher values of total DC (MD ± SE = 67.14 ± 29.16 m; *p* = 0.022, *d* = −0.13) and ACC_<3_ (MD ± SE = 12.33 ± 5.37 counts; *p* = 0.022, *d* = −0.20), but lower values of ACC_>3_ (MD ± SE = −2.23 ± 0.84 counts; *p* = 0.008, *d* = 0.22) in possession games than in position games. WM players reached higher values of ACC_<3_ (MD ± SE = 22.95 ± 6.06 counts; *p* < 0.001, *d* = −0.29) and DEC_<3_ (MD ± SE = 22.96 ± 6.84 counts; *p* < 0.001, *d* = −0.27) in possession games than in position games. F players demonstrated lower values of DEC_>3_ (MD ± SE = −3.56 ± 1.38 counts; *p* = 0.010, *d* = 0.17) in possession games than in position games.

### Medium Dimensions

CD players presented a higher total DC (MD ± SE = 117.05 ± 27.326 m, *p* < 0.001, *d* = −0.27) but demonstrated lower maximal deceleration (MD ± SE = 0.45 ± 0.22 m•s^−2^; *p* = 0.046, *d* = −0.16) during possession games compared to position games. FB and CM players exhibited different outcomes regarding total DC in this game format, with higher demands observed in possession games for FB players (MD ± SE = 115.46 ± 30.22 m; *p* < 0.001, *d* = −0.27) and in position games for CM players (MD ± SE = −97.39 ± 42.43 m; *p* = 0.022, *d* = 0.17). OM players recorded higher ACC_>3_ (MD ± SE = −2.52 ± 1.11 counts; *p* = 0.023, *d* = −0.17) in the position games than in the possession games.

### Large Dimensions

The total DC during possession games was greater than in position games for CD (MD ± SE = 149.68 ± 29.33 m, *p* < 0.001, *d* = −0.34), FB (MD ± SE = 166.34 ± 31.11 m, *p* < 0.001, *d* = −0.39), OM (MD ± SE = 125.05 ± 30.43 m; *p* < 0.001, *d* = −0.30) and F (MD ± SE = 105.38 ± 40.45 m; *p* = 0.009, *d* = −0.18) players. Similarly, peak speed was higher for CM (MD ± SE = 3.00 ± 0.95 km•h^−1^; *p* = 0.002, *d* = −0.25) and F (MD ± SE = 2.14 ± 0.91 km•h^−1^; *p* = 0.019, *d* = −0.19) players in the possession games.

## Discussion

This study examined how pitch size and the task type (positional vs. possession-based games) influenced running demands and external load across different player positions in soccer training. The findings indicate that possession-based games generally imposed greater running demands compared to positional games, particularly on medium and large pitch sizes.

On small pitches, possession-based games elicited higher total DC and more lower-intensity accelerations (ACC_<3_) and decelerations (DEC_<3_). However, they also resulted in lower maximal deceleration, high-intensity accelerations (ACC_>3_) and decelerations (DEC_>3_) when compared to positional games. On larger pitches, possession-based games consistently produced higher peak speeds and overall running demands, demonstrating their utility in replicating higher-intensity actions.

These findings align with a recent study that also identified variations in the external load of position and possession games, although without distinguishing between pitch sizes, reporting higher requirements for ACC_>3_ and DEC_>3_ in position games, as well as greater demands for distance covered, peak speed, and player load in possession games (Asian-Clemente et al., 2024a). Our results reveal a similar trend when comparing position and possession games with different game formats. Specifically, the smallest format used showed the greatest differences between the two tasks, with possession games presenting higher demands for total DC, accelerations, and decelerations of lower intensity, but lower maximum decelerations and high-intensity accelerations and decelerations compared to position games. These results can be explained by the fact that nowadays, it is well established that assigning a specific tactical role imposes different demands on soccer players (Asian-Clemente et al., 2022b), and it has been reported that assigning a tactical role in small-sided games increases demands for high- and maximal-intensity accelerations and decelerations (Asian-Clemente et al., 2024a). Likewise, medium and large formats reported greater distance covered in possession games than in position games, with additional differences in peak speed observed in the largest format. In soccer, players are required to interact with teammates to combat the structure presented by opponents (Memmert, 2015). This interaction is often self-organized (Ribeiro et al., 2019) and conditioned by the contextual information players receive at each moment of the game (Davids et al., 2003), so the fact that a structure established by coaches was not imposed in the possession games could have allowed players to move without specific tactical instructions, leading to higher demands of distance covered and peak speed during these tasks (Asian-Clemente et al., 2024a).

In this study, the positional analysis provides further insight into the specific demands of different players’ roles. Central defenders and fullbacks showed consistently higher running demands during possession-based games, indicating that these formats replicate the broader spatial coverage and sustained activity characteristic of their match-day responsibilities. Conversely, central midfielders demonstrated greater reliance on positional games to simulate their tactical and spatial demands, particularly on medium-sized pitches, where their involvement in orchestrating play is critical. However, possession-based games facilitated higher-speed actions for central midfielders, aligning with scenarios requiring quick transitions or attacking contributions. Wide midfielders and forwards also displayed distinct patterns. Wide midfielders exhibited more low-intensity accelerations and decelerations during possession-based games, aligning with their role in maintaining continuous movement and spatial balance. For forwards, possession-based games on larger pitches encouraged higher speeds and increased running demands, simulating scenarios requiring dynamic offensive play and rapid transitions.

It is well known that the physical demands of players are closely linked to the positions played on the field, as each role involves specific technical and tactical requirements directly associated with distinct physical, physiological, energetic, and biomechanical components (Al Haddad et al., 2018; Asian-Clemente et al., 2022b; Morgans et al., 2024; Riboli et al., 2023). These findings align with previous literature which suggests that soccer imposes position-specific demands not only during matches but also in position and possession games (Asian-Clemente et al., 2024a; Chena et al., 2022; Martin-Garcia et al., 2019; Rabano-Munoz et al., 2024). Similarly, the results are consistent with prior studies indicating that modifications in the playing area influence the demands of position and possession games (Asian-Clemente et al., 2024a, 2025; Chena et al., 2022; Martín-García et al., 2019).

This study demonstrates how the external load of soccer players is influenced by the size and the type of the task performed, as well as the position the player assumes within it. In the literature, it has been extensively studied that CDs are the position with the lowest running demands during matches (Asian-Clemente et al., 2019, 2024b; Suarez-Arrones et al., 2022; Torreño et al., 2016). This could explain why players in this position achieved greater distance covered in all three formats of possession games compared to position games. When performing a tactical task, central defenders tend to adopt a more defensive mindset, focusing primarily on executing defensive tackles, maintaining proper positioning to defend, or intercepting opponents (Modric et al., 2019). These actions involve more frequent changes of speed, which could justify why they exhibited higher maximal deceleration during medium position games. Similarly, previous authors have shown that MFs are the position that covers the greatest distance during matches (Bradley et al., 2010; Di Salvo et al., 2007), which could explain the higher distance covered achieved in the medium-sized position games compared to possession games of the same size. When analyzing the data for FB players, it was observed that they covered greater distance in all three formats of possession games and performed a higher number of ACC_<3_ but fewer DEC_>3_ in the small possession games compared to the small position games. These findings contrast with those reported by other authors when comparing small-sided and position games, since they found differences only in the total sum of accelerations and decelerations >2.5 m•s⁻^2^ in FB players, with higher values observed in the small-sided games (Chena et al., 2022). Methodological differences between studies, as well as variations in the task design (e.g., space, duration, presence of floaters, and inclusion of goals), could explain these discrepancies.

For WM players, among all comparisons made, differences were reported only in DEC_<3_, as more DEC_<3_ were recorded in small possession games. Previously, it has been stated that WMs display the highest and most optimal levels of physical and physiological demands (Torreño et al., 2016). Moreover, they have a linking role in the game, participating in both offensive and defensive phases (Suarez-Arrones et al., 2015). Therefore, their physical fitness and involvement in both tasks could explain the similarity in the external load across both types of tasks. Lastly, OM and F players demonstrated similar patterns, with both showing greater distance covered and higher peak speed during large possession games. Additionally, OM players exhibited more ACC_>3_ during medium position games, while F players displayed more DEC_>3_ during small position games. Considering these specific positions, the findings can be corroborated with previous research by comparing these game tasks (Asian-Clemente et al., 2024a). This reaffirms that there will be higher demands on distance covered and peak speed in possession games, and greater demands on ACC and DEC in position games.

Although this study can contribute to advancing the understanding of small-sided games and their variation with greater tactical application, such as position games, its findings should be interpreted with caution due to several limitations. First, the study was conducted with a single group of players and one specific task format (9 vs. 9 + 2 floaters). Future research should explore other types of position games and include diverse player populations to provide a more comprehensive comparison of these task types. Additionally, it is important to note that the largest format used in this study featured a relative playing area of 115 m^2^ in the position and possession games. Thus, examining these tasks in larger playing spaces would be valuable. Finally, both tasks in this study were carried out with a touch limit constraint. Future investigations should analyze the demands of position games under modified constraints to deepen the understanding of their tactical and physical implications.

## Conclusions

The findings of this study highlight the importance of tailoring small-sided games to the specific tactical and physical objectives of training. Possession-based games are particularly effective for stimulating high-speed running (in the large format) and external load (in terms of distance covered and low-intensity accelerations/decelerations), while positional games, in addition to assigning players specific tactical roles, demand greater high-intensity accelerations/decelerations.

The results show role-specific responses to training formats. Central defenders and fullbacks during possession games are required to cover greater distances regardless of the field size. This aspect does affect other positions, such as central midfielders, who exhibit greater running performance in positional games on medium-sized fields, and offensive midfielders and forwards, who report higher running performance in large-sized possession games. Regarding peak speed, central midfielders and forwards recorded higher values in large possession games compared to position games. In terms of acceleration and deceleration demands, wide midfielders achieved fewer ACC_<3_ and DEC_<3_ events in position games during the small format, while fullbacks recorded fewer ACC_<3_ but more ACC_>3_ in the same format during position games. Coaches should carefully adjust pitch size and the task type to align with the desired training outcomes for different player positions.

### Practical Implications

The insights provided by this research contribute to the design of tasks and planning of soccer training sessions. When setting up their training, coaches need to choose between position and possession games, as well as the size of these games (small, medium, or large). According to the findings of this study, if the aim is to overload high-intensity accelerations and decelerations, position games should be used. However, if the objective is to encourage players to cover more distance and reach higher speeds, large-format possession games are recommended. Additionally, the study demonstrates that incorporating a tactical role into a possession game alters the demands on the entire team as well as on particular positions. Therefore, coaches should not select these tasks based solely on tactical or physical criteria in isolation. Instead, they should consider both aspects simultaneously, with an understanding of the different external load demands that the elements analyzed in this study will impose on players.
